# The Association of Ambient Air Pollution With Cataract Surgery in UK Biobank Participants: Prospective Cohort Study

**DOI:** 10.1167/iovs.62.15.7

**Published:** 2021-12-07

**Authors:** Sharon Y. L. Chua, Anthony P. Khawaja, Parul Desai, Jugnoo S. Rahi, Alex C. Day, Christopher J. Hammond, Peng T. Khaw, Paul J. Foster

**Affiliations:** 1 NIHR Biomedical Research Centre at Moorfields Eye Hospital NHS Foundation Trust & UCL Institute of Ophthalmology, London, United Kingdom; Moorfields Eye Hospital, London; UCL Institute of Ophthalmology, London, United Kingdom; 2 Moorfields Eye Hospital, 162 City Road, London, United Kingdom; 3 UCL Great Ormond Street Institute of Child Health & Institute of Ophthalmology UCL, Holborn, London, London, United Kingdom; 4 Great Ormond Street Hospital NHS Trust, London, United Kingdom; 5 Section of Ophthalmology, School of Life Course Sciences, King's College London, St Thomas’ Hospital, London, United Kingdom

**Keywords:** ambient air pollution, PM_2.5_, cataract surgery, UK Biobank, prospective cohort

## Abstract

**Purpose:**

Air pollution is associated with chronic diseases of later life. Cataract is the most common cause of blindess globally. It is biologically plausible that cataract risk is increased by pollution exposure. Therefore, the relationship between air pollution and incident cataract surgery was examined.

**Methods:**

This was a prospective, observational study involving 433,727 UK Biobank participants. Ambient air pollution measures included particulates, nitrogen dioxide (NO_2_) and nitrogen oxides (NO_x_). Outdoor air pollution was estimated based on land use regression models. Participants undergoing cataract surgery in either eye were ascertained via data linkage to the National Health Service procedure statistics. Those undergoing cataract surgery within 1 year of baseline assessment and those reporting cataract at baseline were excluded. Cox proportional hazards models were used to examine the associations between air pollutants and incident cataract surgery, adjusting for sociodemographic and lifestyle factors.

**Results:**

There were 16,307 incident cases of cataract surgery. Higher exposure to PM_2.5_ was associated with a 5% increased risk of incident cataract surgery (per interquartile range [IQR] increase). Compared to the lowest quartile, participants with exposures to PM_2.5_, NO_2_, and NO_x_ in the highest quartile were 14%, 11%, and 9% more likely to undergo cataract surgery, respectively. A continuous exposure-response relationship was observed, with the likelihood of undergoing cataract surgery being progressively higher with greater levels of PM_2.5_, NO_2_, and NO_x_ (*P* for trend *P* < 0.001).

**Conclusions:**

Although the results of our study showed a 5% increased risk of future cataract surgery following an exposure to PM_2.5_, NO_2_, and NO_x_, the effect estimates were relatively small. Further research is required to determine if the associations identified are causal.

The Global Burden of Diseases, Injuries, and Risk Factors Study (GBD) reports air pollution as a leading cause of disease globally.^1^ The impact is especially pronounced in low- and middle-income countries.^2^ More recently, air pollution has been implicated as a risk factor for chronic eye diseases of later life, including glaucoma, age-related macular degeneration (AMD), adverse structural features in the inner retina, and cataract.^3^^,^^4^ The mechanisms of air pollution-induced health effects are believed to involve oxidative stress.^5^ Oxidative damage may disturb the precise, regular structure of lens proteins, causing an opacity of the crystalline lens.^6^ The majority of those affected are in non-industrialized countries.^7^ Globally, the estimated number of people with severe vision impairment affected by cataract is 78 million and 15 million people are blind because of cataract.^8^ The number of people suffering from cataract is predicted to increase because of an aging population and greater life expectancy. Currently, surgical extraction of the lens is the only available treatment for cataract and access to medical care differs around the world. Thus, identifying modifiable risk factors could help ease the public health burden.

Studies examining the association between air pollution and cataract are few, inconsistent, and European populations have not yet been studied.^3^^,^^9^ There has only been one prospective study that was conducted, and results showed exposure to particulate matter (PM) <10 µm in size (PM_10_) and nitrogen dioxide (NO_2_) were positively associated with incident cataract in the Korean National Insurance Service-National Sample Cohort (NHIS-NSC). In contrast to other studies of the health risks of air pollution, the authors reported there appeared to be a threshold effect in the risk profile. They did not find an association between PM_2.5_ and cataract.^3^

From the known adverse effects of air pollution, it is plausible that it may also be associated with cataract. This analysis therefore explores whether there exists an association between prior exposure to air pollution and risk of future cataract surgery in UK Biobank participants. We used population-wide National Health Service (NHS) activity data to carry out the largest longitudinal study to date, with the aim of confirming and characterizing a possible relationship between pollution and visually significant cataract, using cataract surgery as a surrogate outcome.

## Methods

### Study Population

The UK Biobank is a very large multisite community-based cohort study consisting of UK residents aged 40 to 69 years at enrollment. Participants who were registered with the NHS and lived within 25 miles of any of the 22 assessment centers were invited to join the study. Baseline examinations consisting of questionnaires and measurements were carried out between 2006 and 2010.^10^ Eye measurements were collected in late 2009 in 6 assessment centers (5 in England and 1 in Wales) as an additional enhancement to the initial baseline assessment.^11^ The North West Multi-center Research Ethics Committee approved the study in accordance with the principles of the Declaration of Helsinki. The overall study protocol (http://www.ukbiobank.ac.uk/resources/) and protocols for individual tests (http://biobank.ctsu.ox.ac.uk/crystal/docs.cgi) are available online. Participants answered a detailed touch-screen questionnaire covering demographic, socioeconomic, lifestyle, and systemic and ocular diseases information, including their cataract status. Townsend deprivation index was determined using participants’ postcodes at recruitment and the corresponding output areas from the preceding national census. Based on the output area's employment status, home and car ownership, and home condition, the index was calculated. A higher and more positive index value represented a more deprived area. Smoking status was determined by self-reported history of smoking tobacco in the past or those who were currently smoking at baseline. Definition of diabetes mellitus (DM) included self-reported type 1 or type 2 diabetes and self-reported use of insulin. Definition of hypertension included self-reported hypertension. Physical measures included blood pressure, height, and weight.^10^ Body mass index (BMI) was defined as weight (kg) divided by height (m) squared. Ocular measurements commenced in late 2009 at 6 assessment centers as an additional enhancement to the initial baseline measures; detailed methods have been published.^11^ As part of the ocular assessment, self-reported eye diseases, including glaucoma, diabetes related eye disease, and AMD, were obtained from self-administered questionnaires. Refractive error was measured with an autorefractor (Tomey RC5000, Nagoya, Japan) and spherical equivalent refraction (SER) was calculated as sphere power plus half cylinder power.

### Air Pollution Measurement

Air pollution measures were provided by the Small Area Health Statistics Unit (http://www.sahsu.org/) as part of the BioSHaRE-EU Environmental Determinants of Health Project (http://www.bioshare.eu/), and were linked centrally to the assessment data by UK Biobank analysts (http://biobank.ctsu.ox.ac.uk/crystal/docs/Enviro-ExposEst.pdf). Detailed measures of air pollutants have been published.^12^^,^^13^ The annual average concentrations of PM ≤2.5 µm in aerodynamic diameter (PM_2.5_), PM_2.5–10_ (aerodynamic diameter between 2.5 and 10 µm), PM_10_ (aerodynamic diameter of less than 10 µm), PM_2.5_ ab (PM_2.5_ absorbance) a measurement of the blackness of PM_2.5_ filter – a proxy for elemental or black carbon, NO_2_, and nitrogen oxides (NO_x_) were calculated centrally by UK Biobank using a land use regression model developed by the European Study of Cohorts for Air Pollution Effects (ESCAPE) project (http://www.escapeproject.eu/).^14^ The model uses a range of Geographic Information System–derived predictor variables, such as traffic intensity, population, topography, and land use, to calculate the annual average air pollution concentration at participants’ baseline residential addresses. NO_2_ annual concentration data were available for four years (2005, 2006, 2007, and 2010), whereas PM_10_ data was available for 2007 and 2010. These values were averaged to obtain the mean estimate. All other PM and nitrogen pollutants had the exposure data for a single year (2010).

### Ascertainment of Incident Cataract Surgery

Incident cataract surgery was determined via linkage to hospital procedure records, namely Hospital Episode Statistics (HES) for England, Scottish Morbidity Record (SMR) for Scotland, and the Patient Episode Database for Wales (PEDW). Participants with an OPCS Classification of Interventions and Procedures (OPCS-4) code of C71.2 – “Phacoemulsification of lens” or C75.1 – “Insertion of prosthetic replacement for lens” were determined to have had cataract surgery, using the date of (first eye) surgery as the date of event. Participants with cataract surgery within 1 year after the baseline assessment visit were excluded to reduce the chance of prevalent visually significant cataract being present at baseline. We excluded those with cataract surgery in 2010 or earlier as air pollution measurements were collected between 2005 and 2010. Participants with self-reported cataract at baseline were also excluded from this study.

### Statistical Analysis

The baseline characteristics of participants between those with and without incident cataract surgery were compared. Descriptive statistics for continuous variables are presented as mean (standard deviation [SD]), whereas categorical variables are presented as number (percentage). A survival analysis was performed, and participants were censored at the following end points: date of death, or end of the data linkage (March 31, 2017), whichever came first. Cox proportional hazards models were used to examine associations of each air pollutant with incident cataract surgery, and the proportional hazards assumption was met. All associations were examined using two multivariable models. Model 1 was adjusted for age and sex, whereas model 2 was additionally adjusted for race, Townsend deprivation index, BMI, smoking, and diabetes status. In a sensitivity analysis, ocular factors including SER, self-reported glaucoma, AMD, and diabetes related eye disease were additionally adjusted for in the multivariable models due to its association with cataract risk. Ocular factors were not included in the primary analysis given the data was only available for a sub-sample (*N* = 105,182); eye measures were only included in the later phases of the UK Biobank phenotyping effort. The effect estimates represent the risk of incident cataract surgery per interquartile range (IQR) or quartile increment in air pollutant. Statistical significance was set at *P* < 0.05. Data analysis was performed using STATA software (version 16; StataCorp LP, College Station, TX, USA).

## Results

**Figure 1.**
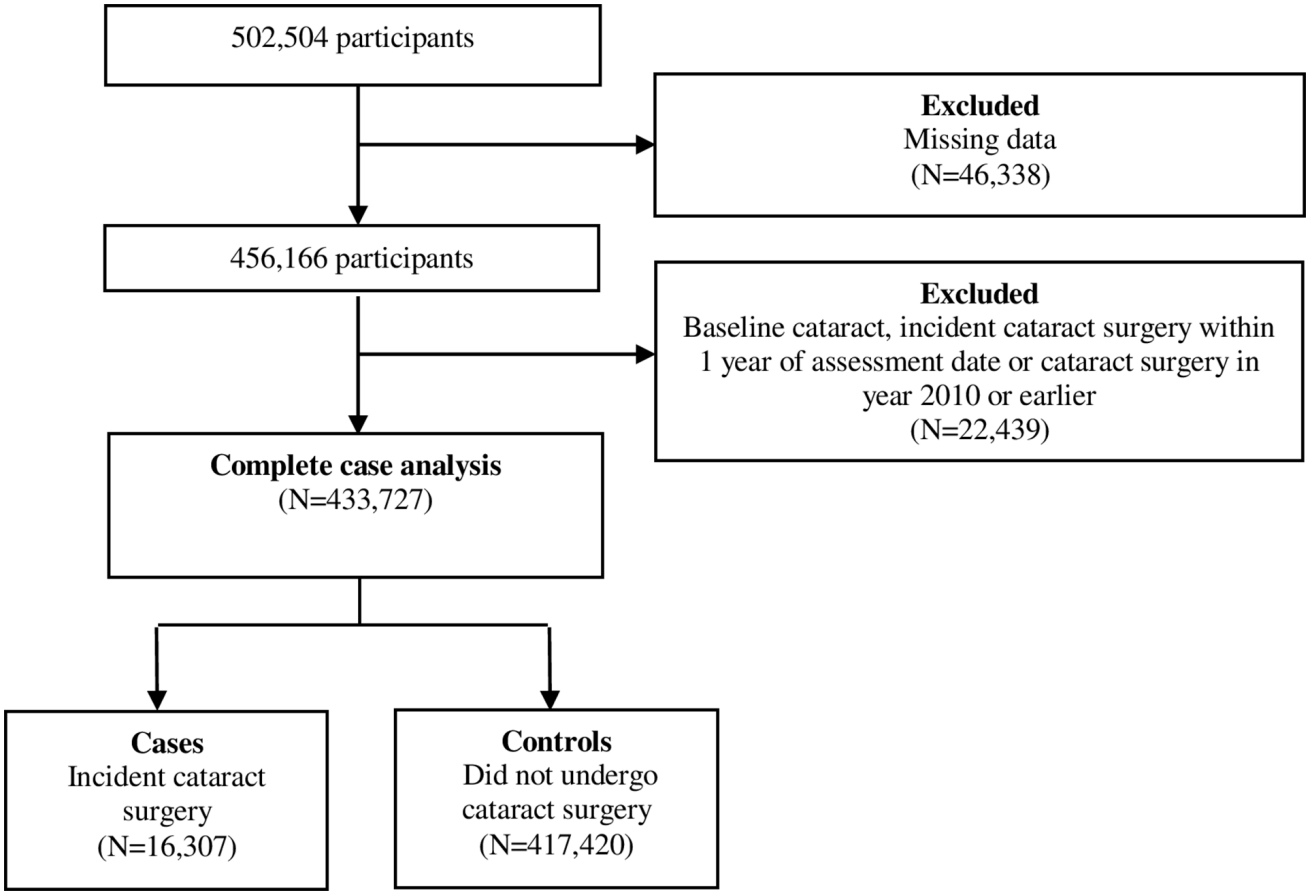
Flowchart of participants included in the UK Biobank cohort.

Of the 502,504 UK Biobank participants, 433,727 participants were included following the exclusion of 46,338 participants with missing data and 22,439 participants with baseline cataract, incident cataract surgery within 1 year, or cataract surgery in 2010 or earlier (Fig. 1). The mean follow-up time was 94 months (SD = 15 months) during which time 16,307 (3.8% of the total) participants underwent cataract surgery. Table 1 presents the characteristics of participants included in this study. Compared with controls, participants who had undergone cataract surgery were older, more likely to be women, non-White, more likely to reside in a more deprived area, have a higher BMI, more likely to have ever smoked, and have diabetes (all *P* < 0.001). Compared with participants who were included, those excluded were older, more likely women, and more likely to reside in a more deprived area, have a higher BMI, more likely to have ever smoked, and have diabetes (all *P* < 0.001; Supplementary Table S1). Table 2 shows the distribution of ambient air pollutants, with the median concentrations higher for NO_2_ and NO_x_ than for PM.

**Table 1.** Baseline Characteristics of Participants Included in the Study, According to Incident Cataract Surgery Status

	Mean (SD)/*n* (%)		
	Incident Cataract Surgery (*N* = 16,307)	Control (*N* = 417,420)	*P* Value
Age (years)	62.5 (5.6)	56.0 (8.1)	<0.001
Sex			<0.001
Men	6,993 (42.9)	191,483 (45.9)	
Women	9,314 (57.1)	225,937 (54.1)	
Race			<0.001
White	15,174 (93.1)	395,258 (94.7)	<0.001
Non-White	1,133 (6.9)	22,162 (5.3)	
Townsend deprivation index	−1.2 (3.1)	−1.3 (3.0)	<0.001
Body mass index (kg/m^2^)	27.9 (4.9)	27.4 (4.8)	<0.001
Smoking status			<0.001
Never	8,063 (49.4)	230,036 (55.1)	
Ever smoke	8,244 (50.6)	187,384 (44.9)	
Diabetes status			<0.001
Non-diabetic	14,524 (89.1)	397,633 (95.3)	
Diabetic	1,783 (10.9)	19,787 (4.7)	

Mean (SD) is presented for continuous variables and count (%) is presented for categorical variables.

SD, standard deviation.

**Table 2.** Distribution of Ambient Air Pollutants

	Median (IQR)	Range
PM_2.5_ (µg/m^3^)	9.93 (1.27)	(8.17 to 21.31)
Quartile 1	8.79	(8.17 to 9.29)
Quartile 2	9.64	(9.30 to 9.93)
Quartile 3	10.22	(9.94 to 10.56)
Quartile 4	11.13	(10.57 to 21.31)
PM_2.5_ absorbance (µg/m^3^)	1.13 (0.30)	(0.83 to 4.60)
Quartile 1	0.93	(0.83 to 1.00)
Quartile 2	1.07	(1.01 to 1.13)
Quartile 3	1.21	(1.14 to 1.30)
Quartile 4	1.46	(1.31 to 4.60)
PM_2.5–10_ (µg/m^3^)	6.11 (0.80)	(5.57 to 12.82)
Quartile 1	5.72	(5.57 to 5.84)
Quartile 2	5.97	(5.85 to 6.11)
Quartile 3	6.31	(6.12 to 6.64)
Quartile 4	7.30	(6.65 to 12.82)
PM_10_ (µg/m^3^)	19.14 (2.33)	(12.86 to 30.52)
Quartile 1	17.22	(12.86 to 18.06)
Quartile 2	18.63	(18.07 to 19.14)
Quartile 3	19.70	(19.15 to 20.39)
Quartile 4	21.55	(20.40 to 30.52)
Nitrogen dioxide (µg/m^3^)	28.03 (10.95)	(8.86 to 125.12)
Quartile 1	19.53	(8.86 to 22.91)
Quartile 2	25.60	(22.92 to 28.03)
Quartile 3	30.67	(28.04 to 33.85)
Quartile 4	39.16	(33.86 to 125.12)
Nitrogen oxides (µg/m^3^)	42.20 (16.55)	(19.74 to 265.94)
Quartile 1	28.07	(19.74 to 34.16)
Quartile 2	38.43	(34.17 to 42.20)
Quartile 3	46.04	(42.21 to 50.71)
Quartile 4	58.64	(50.72 to 265.94)

IQR, Interquartile range; PM_2.5_, Particulate matter less than 2.5 µm in aerodynamic diameter; PM_2.5_ ab, (PM_2.5_ absorbance) a measurement of the blackness of PM2.5 filter - a proxy for elemental or black carbon; PM_2.5–10_, particulate matter between 2.5 µm and 10 µm in aerodynamic diameter and PM_10_, particulate matter less than 10 µm in aerodynamic diameter.

**Figure 2.**
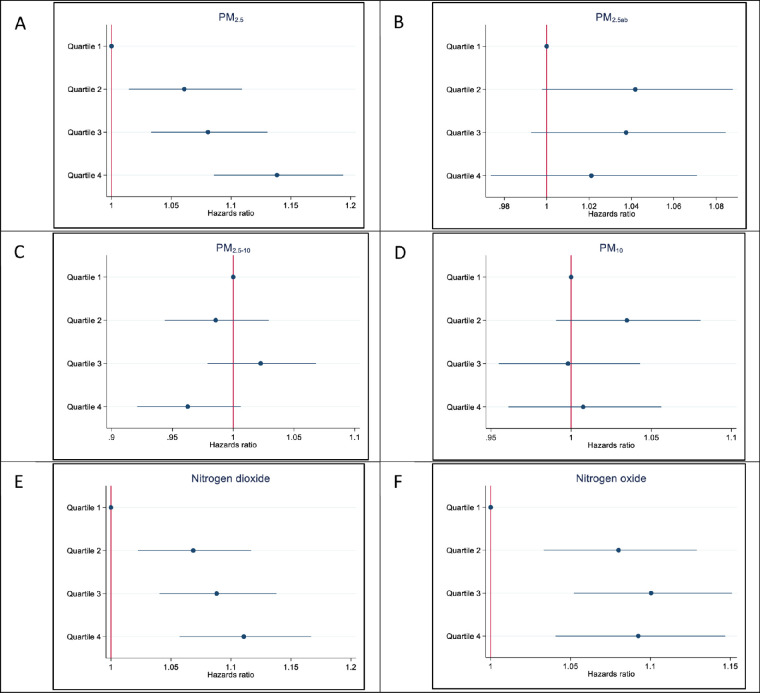
**Multivariable hazards ratio of incident cataract surgery per quartile increase in ambient air pollution.** Adjusted for age, sex, race, Townsend deprivation index, body mass index, smoking status, and diabetes. PM_2.5_, particulate matter less than 2.5 µm in aerodynamic diameter; PM_2.5_ ab, (PM_2.5_ absorbance) a measurement of the blackness of PM_2.5_ filter - a proxy for elemental or black carbon; PM_2.5–10_, particulate matter between 2.5 µm and 10 µm in aerodynamic diameter, and PM_10_, particulate matter less than 10 µm in aerodynamic diameter.

After adjusting for age and sex, higher exposure to all types of ambient air pollutants, except PM_2.5–10_, were associated with higher risk of incident cataract surgery (*P* < 0.001; Table 3). In the multivariable model, after adjusting for age, sex, race, Townsend deprivation index, BMI, smoking, and diabetes status, greater exposure to PM_2.5_ was the most strongly associated with a 5% increased risk of incident cataract surgery (hazard ratio [HR] = 1.05, 95% confidence interval [CI] = 1.03 to 1.07, per IQR increase). Likewise, the risk of incident cataract surgery increased by 4% (HR = 1.04, 95% CI = 1.01 to 1.06, per IQR increase), and 3% (HR = 1.03, 95% CI = 1.01 to 1.05, per IQR increase) after exposure to higher levels of NO_2_ and NO_x_, respectively. The risk of incident cataract surgery was progressively higher with greater exposure to PM_2.5_, NO_2_, and NO_x_ (*P* for trend < 0.001; see Table 3 and Fig. 2). Compared to ambient air pollution in the first quartile, exposure to PM_2.5_, NO_2_, and NO_x_ in the highest quartile had 14%, 11%, and 9% higher risk of incident cataract surgery, respectively. In contrast, exposure to PM_2.5_ ab, PM_2.5–10_, and PM_10_ were not associated with incident cataract surgery in fully adjusted models. When we combined PM_2.5_ and NO_x_ in one model, PM_2.5_ was significantly associated with a 6% increased risk of future cataract surgery (HR = 1.06, 95% CI = 1.03 to 1.08, per IQR increase), while NO_x_ was not associated with future risk of cataract surgery (HR = 1.00, *P* = 0.18). After the additional adjustment for hypertension status, the risk of incident cataract surgery increased by 5% (HR = 1.04, 95% CI = 1.03 to 1.07, per IQR increase), 3% (HR = 1.03, 95% CI = 1.01 to 1.05, per IQR increase), and 4% (HR = 1.03, 95% CI = 1.02 to 1.06, per IQR increase) after exposure to higher levels of PM_2.5_, NO_x_, and NO_2_ respectively.

**Table 3.** Multivariable Associations of Ambient Air Pollution With Incident Cataract Surgery (*n* = 433,727)

	Age and Sex Adjusted		Multivariable Model	
	HR (95% CI)	*P* Value	HR (95% CI)	*P* Value
**Ambient air pollution (µg/m^3^)**				
PM_2.5_ (per IQR increase)	1.13 (1.11 to 1.15)	**<0.001**	1.05 (1.03 to 1.07)	**7.5 × 10^−^^7^**
First quartile	Ref		Ref	
Second quartile	1.11 (1.06 to 1.16)	**<0.001**	1.06 (1.01 to 1.11)	**0.009**
Third quartile	1.17 (1.12 to 1.22)	**<0.001**	1.08 (1.03 to 1.13)	**0.001**
Fourth quartile	1.33 (1.27 to 1.39)	**<0.001**	1.14 (1.08 to 1.19)	**8.7 × 10^−^^8^**
*P* for trend		**<0.001**		**1.2 × 10^−^^7^**
PM_2.5_ absorbance (per IQR increase)	1.08 (1.06 to 1.10)	**<0.001**	1.00 (0.99 to 1.02)	0.53
First quartile	Ref		Ref	
Second quartile	1.08 (1.04 to 1.13)	**<0.001**	1.04 (1.00 to 1.09)	0.06
Third quartile	1.13 (1.08 to 1.18)	**<0.001**	1.04 (0.99 to 1.08)	0.10
Fourth quartile	1.24 (1.18 to 1.29)	**<0.001**	1.02 (0.97 to 1.07)	0.59
*P* for trend		**<0.001**		0.38
PM_2.5–10_ (per IQR increase)	1.00 (0.99 to 1.02)	0.55	0.99 (0.97 to 1.00)	0.05
First quartile	Ref		Ref	
Second quartile	1.01 (0.97 to 1.06)	0.51	0.99 (0.94 to 1.03)	0.51
Third quartile	1.09 (1.05 to 1.14)	**<0.001**	1.02 (0.98 to 1.07)	0.32
Fourth quartile	1.05 (1.01 to 1.10)	**0.024**	0.96 (0.92 to 1.01)	0.09
*P* for trend		**0.001**		0.27
PM_10_ (per IQR increase)	1.10 (1.07 to 1.12)	**<0.001**	1.00 (0.99 to 1.02)	0.94
First quartile	Ref		Ref	
Second quartile	1.08 (1.03 to 1.12)	**0.001**	1.03 (0.99 to 1.08)	0.12
Third quartile	1.07 (1.03 to 1.12)	**0.001**	1.00 (0.96 to 1.04)	0.93
Fourth quartile	1.21 (1.16 to 1.26)	**<0.001**	1.01 (0.96 to 1.06)	0.76
*P* for trend		**<0.001**		0.84
Nitrogen dioxide (per IQR increase)	1.14 (1.12 to 1.16)	**<0.001**	1.04 (1.01 to 1.06)	**0.001**
First quartile	Ref		Ref	
Second quartile	1.11 (1.06 to 1.16)	**<0.001**	1.07 (1.02 to 1.12)	**0.003**
Third quartile	1.17 (1.12 to 1.22)	**<0.001**	1.09 (1.04 to 1.14)	**2.1 × 10^−^^4^**
Fourth quartile	1.36 (1.30 to 1.42)	**<0.001**	1.11 (1.06 to 1.17)	**3.0 × 10^−^^5^**
*P* for trend		**<0.001**		**7.2 × 10^−^^4^**
Nitrogen oxides (per IQR increase)	1.10 (1.08 to 1.11)	**<0.001**	1.03 (1.01 to 1.05)	**0.001**
First quartile	Ref		Ref	
Second quartile	1.13 (1.08 to 1.18)	**<0.001**	1.08 (1.03 to 1.13)	**6.4 × 10^−^^4^**
Third quartile	1.20 (1.15 to 1.26)	**<0.001**	1.10 (1.05 to 1.15)	**3.0 × 10^−^^5^**
Fourth quartile	1.31 (1.25 to 1.37)	**<0.001**	1.09 (1.04 to 1.15)	**3.6 × 10^−^^4^**
*P* for trend		**<0.001**		**2.2 × 10^−^^4^**

The hazards ratio represents per IQR increase in exposure variable.

Adjusted for age, sex, race, Townsend deprivation index, body mass index, smoking status, and diabetes.

IQR, interquartile range; PM_2.5_, particulate matter less than 2.5 µm in aerodynamic diameter; PM_2.5_ ab, (PM2.5 absorbance) a measurement of the blackness of PM2.5 filter - a proxy for elemental or black carbon; PM_2.5–10_, particulate matter between 2.5 µm and 10 µm in aerodynamic diameter, and PM_10_, particulate matter less than 10 µm in aerodynamic diameter.

In sensitivity analysis, after additional adjustment for SER, self-reported glaucoma, AMD, and diabetes related eye disease, the overall trends of the association between ambient air pollution and incident cataract surgery remained and are shown in Supplementary Table S2. In this smaller group (*N* = 105,182), there was a significant association between increasing exposure to PM_2.5_ and higher risk of incident cataract surgery (*P* for trend = 0.047). Compared to PM_2.5_ in the lowest quartile, exposure to PM_2.5_ in the highest quartile had 13% higher risk of incident cataract surgery (HR = 1.13, 95% CI = 1.01 to 1.27). Exposure to PM_10_ in the third quartile was associated with a 13% increased risk of cataract surgery compared to the lowest quartile.

## Discussion

In this large study of UK Biobank participants, we have identified higher baseline ambient PM_2.5_, NO_2_, and NO_x_ levels to be associated with a higher risk of undergoing subsequent cataract surgery. A dose-response relationship was also observed between higher levels of air pollutants and increased risk of cataract surgery. We found the highest risk was related to levels of PM_2.5_. This suggests that long-term exposure to ambient air pollution, particularly smaller particulates and combustion-related pollutants, may increase the risk of cataract formation requiring surgery in the older adult population.

Previous studies examining the association between exposure to air pollution and cataract have shown inconsistent findings and have been limited by their cross-sectional design, smaller sample size, and have only been examined in the Asian populations.^9^^,^^15^^,^^16^ In a cross-sectional study of 5871 participants, a 1-SD increase in years of biomass fuel use was associated with a 28% increase in nuclear cataract for women in the Indian Eye Study, respectively.^16^ In contrast, the KNHANES of 18,622 participants reported that higher ozone concentrations was associated with lower odds of cataract, whereas no significant association was identified between PM_10_ and NO_2_ with cataract.^9^ There has only been one longitudinal study that examined the relationship between ambient air pollution and cataract.^3^ In a prospective Korean cohort (NHIS-NSC) of 115,728 participants, higher exposures to PM_10_, NO_2_, and SO_2_ were associated with higher risk of cataract, whereas there was no significant association between PM_2.5_ and incident cataract. Our study is longitudinal in design and the largest to date, to the best of our knowledge. Furthermore, we used NHS activity data to define cataract surgery and were able to capture population-wide UK residents, as the NHS provides healthcare to all UK residents.

Outdoor air pollution was estimated using the participants’ home address and does not capture any exposure to indoor pollutants. According to the Environmental Protection Agency, the levels of air pollution may be two to five times higher indoors than outdoors.^17^ Clearly, the particulate levels at the home address does not give a completely robust measure of the habitual, ambient exposure to, and impact of, all pollutants. Therefore, our primary exposure measure is imperfect and is likely that our risk estimates may have been attenuated.

Our results showed that greater baseline ambient PM_2.5_, NO_2_, and NO_x_ exposure were associated with higher risk of future cataract surgery. Furthermore, there was a dose-response relationship of progressively likelihood of cataract surgery with in people exposed to higher levels of PM_2.5_, NO_2_, and NO_x_. No significant associations were observed for PM_2.5–10_ and PM_10_. Although the results of our study showed a 5% increased risk of future cataract surgery following an exposure to PM_2.5_, NO_2_, and NO_x_, the effect estimates were relatively small within the range of air pollutants seen in this study. Ambient air pollution increases oxidative stress by generating free radicals^5^ and long-time exposure of oxidative stress leads to accumulation of damaged lens proteins, leading to cataract formation.^18^ Furthermore, lens antioxidants, including glutathione (GSH) and ascorbate, which protect the nuclear lens protein from the effects of reactive oxygen species, are depleted following exposure to biomass fuels.^19^ Previous studies have examined the effectiveness of antioxidants to prevent or slow the progression of cataracts. A Cochrane review did not find evidence from randomized controlled trials (RCTs) that supplementation with antioxidant vitamins, including beta-carotene, vitamin C, or vitamin E, prevent or slow the progression of age-related cataract.^20^ It is possible that the natural history of cataract is so long that anything other than very large RCTs will detect a beneficial effect. In comparison with these RCTs, our cohort study had a longer follow-up period (mean = 7.8 years) and a much larger sample size. Air pollutants including NO_x_ (nitric oxide and NO_2_) and PM_2.5_ particles are mainly formed during combustion processes and high levels of respirable particulates from biomass fuel, especially PM_2.5_, have been reported in India.^21^ The adverse health effects observed by fine PM (PM_2.5_) compared with coarse PM (PM_2.5–10_) may be explained by the absorption of fine PM into the bloodstream through alveolar capillaries causing systemic inflammation.^22^ Fine PM mainly result from combustion processes and combustion-related particles are known to be more toxic to health, causing airway and systemic inflammation and myocardial ischemia, compared with particles not generated by combustion.^23^

The primary outcome in our study was cataract surgery, which is a surrogate for visually significant cataract. It is possible that other factors, including access to healthcare and the presence of other eye diseases, may also influence whether a person undergoes cataract surgery. We found that adjustment for other eye diseases attenuated the association between air pollution and further cataract surgery. In our previous findings,^24^^–^^26^ participants exposed to higher levels of air pollutants were at greater risk of developing glaucoma, AMD, and adverse changes in the retinal structures. Furthermore, a recent finding by the Canadian Longitudinal Study on Aging consisting of 30,097 adults aged 45 to 85 years reported increased PM_2.5_ was associated with glaucoma.^27^ As the retina (and especially the outer retina) has a very rich vascular supply, it may be that the delivery of blood-borne pollutants reach the retina in significantly higher concentrations, giving more direct adverse effects. The lens receives oxygen and nutrients via the aqueous humour (a secretion product of the blood), which also contains ascorbic acid at a concentration 20 times that of serum.^28^ We hypothesize that the dilution of oxidative stressors in the aqueous, and the counter-balancing effect of ascorbic acid, reduce the impact of pollutants on the lens, compared to impact on the retina and optic nerve. Therefore, the additional adjustment for AMD and glaucoma may be masking the effect of the pollutants on the more protected lens.

It is possible that visual symptoms from other chronic age-related eye diseases (primarily AMD, as it is the most common, and the most symptomatic) may lead to presentation to eye care services, and this results in cataract surgery. However, data from The Beaver Dam Longitudinal Eye Study found that cataract in the presence of signs of early AMD is no more likely to lead to subsequent cataract surgery than in the absence of early AMD. In addition, the same investigators found that eyes with cataract at baseline who had developed incident early AMD 5 years later were no more likely to have poorer visual acuity at baseline than eyes with cataract without incident AMD. There is an increased risk of incident AMD after cataract surgery, leading some to suggest that older models of intraocular lens implants allow greater transmission of higher energy light, causing retinal oxidative stress, leading to AMD. Thus, cataract formation seems to precede AMD.^29^

Although the PM_2.5_ and NO_2_ concentrations in our analyses were within the World Health Organization (WHO) ambient air quality guidelines of annual means of 10 µg/m^3^ and 40 µg/m^3^, respectively, we detected a 6% and 3% increased risk of incident cataract surgery per IQR increase in PM_2.5_ and NO_2_, respectively. According to the WHO estimates, the air pollution levels exceed recommended limits in 98% of cities in low- and middle-income countries.^30^ In cities in non-industrialized countries, air pollution is much higher due to high population densities and lower regulatory standards. High annual mean PM_2.5_ concentrations have been reported in India (138 µg/m^3^), China (138 µg/m^3^), and Nepal (262 µg/m^3^) and Niger (213 µg/m^3^).^31^ These levels of pollution have great implications for health, given the large populations exposed to them. Although there has been a reduction in pollution in Chinese cities, the levels still remain high despite strong policy measures. Industry and vehicles are the most likely source of ambient PM_2.5_ in our dataset. However, household air pollution (from cooking and heating fuels) may cause even higher levels of exposure and morbidity. Household indoor air pollution appears particularly prevalent in low and middle income countries. Although there have been falls in PM_2.5_ and NO_2_ concentrations across all continents during the coronavirus disease 2019 (COVID-19) lockdown, with the greatest reductions in India, it is likely these will rise again as economies accelerate. Cataract surgery is not always readily available in developing countries and loss of vision or visual impairment resulting from cataract is of great socioeconomic importance. Because ambient air pollution is also related to other degenerative ocular diseases,^24^^–^^26^ strategies to reduce the levels of air pollution offer a novel intervention to control chronic eye diseases of later life. In the United Kingdom, the guidelines for referring a person for cataract surgery is dependent on how the cataract affects the person's vision and quality of life.^32^ This is similar to the United States, where cataract surgery is recommended when there is a lens opacity that imposes functional impairment.^33^^,^^34^

Strengths of our study include its longitudinal design with long-term follow up and the very large sample size, which provides unprecedented statistical power to examine the relationship between ambient air pollution and cataracts. Limitations of the study include that UK Biobank has similarities to a “healthy volunteer” selection bias, and participants are likely healthier than the general population. UK Biobank participants are comparatively young (mean = 56 years old) and not many would have reached the age to require cataract surgery. Therefore, it is likely that the proportion of incident cataract surgery may be higher in the general population and the risks that we have calculated may have been underestimated. Cases of cataract were identified by linkage to routine NHS surgical activity data. There was no information on cataract subtype in our cohort. Thus, we were unable to examine the association of ambient air pollution on the different types or severity of cataract. As cataract is a slow developing process, we are unable to rule out the possibility that cataract development may have preceded our exposure assessment of air pollution measures. However, cataract surgery is a hard end point, and it followed the exposure measurement timepoint. Our findings do allow us to understand the relationship between cataract that was severe enough to cause visual symptoms, which required surgical intervention, and air pollution. Cataract surgery is the most commonly performed major operation in the United Kingdom. It is typically carried out to improve vision in the setting of visually significant, age-related lens opacity, although it is sometimes carried out for other conditions, such as to achieve improved intraocular pressure control in glaucoma.

In summary, this is the largest prospective study to examine the relationship between prior exposure to ambient air pollutants and the need for future cataract surgery. Our study suggests exposure to air pollution may correlate with an increased risk of an individual undergoing cataract surgery, a proxy for visually significant cataract.

## Supplementary Material

Supplement 1
