# Supplementary material for: The Association of Ambient Air Pollution With Cataract Surgery in UK Biobank Participants: Prospective Cohort Study
Source: Invest Ophthalmol Vis Sci. 2021 Dec 7;62(15):7. doi: 10.1167/iovs.62.15.7 (PMC8662572; doi:10.1167/iovs.62.15.7)
Supplement: Supplement 1 [file iovs-62-15-7_s001.pdf]

Table S1. Comparison of baseline characteristics of UK Biobank participants included and excluded from the study

	Included	Excluded	*P*-value
Sample size	433,727	68,777	
Age (years), mean (SD)	56.3 (8.1)	58.1 (8.1)	<0.001
Sex, n (%)			<0.001
Men	198,476 (45.8)	30,646 (44.6)	
Women	235,251 (54.2)	38,131 (55.4)	
Ethnicity, n (%)			0.49
White	410,432 (94.6)	65,039 (94.6)	
Non-white	23,295 (5.4)	3,738 (5.4)	
Townsend deprivation index, mean (SD)	-1.4 (3.0)	-0.9 (3.4)	<0.001
Body mass index (kg/m^2), mean (SD)	27.4 (4.8)	27.6 (4.9)	<0.001
Smoking status, n (%)			<0.001
Never smoked	238,099 (54.9)	35,423 (53.8)	
Ever smoked	195,628 (45.1)	30,406 (46.2)	
Diabetes status, n (%)			<0.001
No	412,157 (95.0)	63,674 (92.6)	
Yes	21,570 (5.0)	5,103 (7.4)	

In UK Biobank, 623 people had missing Townsend deprivation index data, 3,105 people had missing BMI data and 2,948 people had missing smoking status data.

Table S2: Multivariable associations of ambient air pollution with incident cataract surgery, with additional adjustment for ocular factors (n=105,182)

	**Multivariable model**	
	HR (95% CI)	*P*-value
**Ambient air pollution (μg/m^3)**		
$PM_{2.5}$ (per IQR increase)	1.05 (0.99, 1.10)	0.09
First quartile	Ref	
Second quartile	1.05 (0.95, 1.16)	0.34
Third quartile	1.05 (0.95, 1.16)	0.36
Fourth quartile	1.13 (1.01, 1.27)	**0.030**
P for trend		**0.047**
$PM_{2.5}$ absorbance (per IQR increase)	1.00 (0.96, 1.04)	0.99
First quartile	Ref	
Second quartile	1.10 (0.98, 1.24)	0.10
Third quartile	1.03 (0.92, 1.16)	0.55
Fourth quartile	0.99 (0.88, 1.10)	0.85
P for trend		0.36
$PM_{2.5\text{-}10}$ (per IQR increase)	1.00 (0.96, 1.03)	0.96
First quartile	Ref	
Second quartile	1.09 (0.98, 1.22)	0.12
Third quartile	1.03 (0.93, 1.15)	0.54
Fourth quartile	1.06 (0.94, 1.18)	0.33
P for trend		0.68
PM_{10} (per IQR increase)	1.03 (0.99, 1.08)	0.16
First quartile	Ref	
Second quartile	1.03 (0.92, 1.15)	0.58
Third quartile	1.13 (1.01, 1.27)	**0.041**
Fourth quartile	1.10 (0.99, 1.23)	0.09
P for trend		**0.042**
Nitrogen dioxide (per IQR increase)	1.01 (0.96, 1.06)	0.61
First quartile	Ref	
Second quartile	1.09 (0.96, 1.24)	0.16
Third quartile	1.11 (0.98, 1.25)	0.09
Fourth quartile	1.09 (0.97, 1.23)	0.14
P for trend		0.24
Nitrogen oxide (per IQR increase)	1.00 (0.96, 1.05)	0.82
First quartile	Ref	
Second quartile	1.07 (0.96, 1.19)	0.21
Third quartile	1.07 (0.96, 1.19)	0.22
Fourth quartile	1.02 (0.90, 1.15)	0.77
P for trend		0.89

Adjusted for age, sex, race, Townsend deprivation index, body mass index, smoking status, diabetes, spherical equivalent refraction, self-reported glaucoma, age-related macular degeneration and diabetes related eye disease.

IQR, interquartile range; $PM_{2.5,}$ Particulate matter less than 2.5 μm in aerodynamic diameter (μg/m^3); $PM_{2.5}$ ab, (PM2.5 absorbance) a measurement of the blackness of PM2.5 filter - a proxy for elemental or black carbon; $PM_{2.5\text{-}10,}$ Particulate matter between 2.5 μm to 10 μm in aerodynamic diameter (μg/m^3) and PM_{10}, Particulate matter less than 10 μm in aerodynamic diameter (μg/m^3)
